# Pseudomonas Keratitis: From Diagnosis to Successful Deep Anterior Lamellar Keratoplasty

**DOI:** 10.7759/cureus.56154

**Published:** 2024-03-14

**Authors:** Kirupakaran Arun, Panagiotis Georgoudis

**Affiliations:** 1 Ophthalmology, Whipps Cross Hospital, London, GBR

**Keywords:** pseudomonas keratitis, deep anterior lamellar keratoplasty, corneal graft, bacterial keratitis, keratitis, refractory corneal ulcer

## Abstract

Pseudomonas keratitis is an aggressive form of bacterial keratitis that can have devastating consequences, such as corneal perforation, if not promptly identified and appropriately managed. The aim of this case report is to highlight key clinical features of Pseudomonas keratitis and evaluate the initial and long-term management options for this condition. We report a case of a 32-year-old female who presented with a large corneal abscess and hypopyon following contact lens wear. Corneal cultures confirmed Pseudomonas as the causative organism and she was treated with topical levofloxacin and gentamycin. Following sterilisation of the corneal ulcer, the patient was left with deep stromal scarring, peripheral corneal thinning as well as four-quadrant deep corneal vascularisation. She was listed for deep anterior lamellar keratoplasty surgery to clear her visual axis. We highlight some of the challenges that were faced both intra-operatively and post-operatively and how they were managed.

## Introduction

Microbial keratitis is one of the most common presentations to the eye emergency department and is responsible for approximately one million healthcare appointments per year in the United States [1]. Bacterial keratitis is the most common type of microbial keratitis [1] and is an ophthalmic emergency that requires urgent attention due to its rapid progression [2].

The major risk factor for bacterial keratitis is contact lens wear [3], and it is essential to ask about contact lens use and hygiene in any patient with signs suggestive of microbial keratitis. The main features of bacterial keratitis seen on clinical examination include round corneal ulceration [3], white stromal infiltrates with distinct borders [4], and if severe, the presence of a hypopyon [4].

All corneal ulcers should be cultured to identify the causative organism and antibiotic sensitivities enable optimisation of antibiotic therapy. Polymerase chain reaction should also be used as it is more sensitive [5] and generally provides faster results.

Once the corneal ulcer has been sterilised with appropriate topical antibiotic therapy, the patient may require surgical management to optimise vision due to corneal scarring secondary to bacterial keratitis. The two main surgical options for clearing the visual axis in patients with stromal scarring are penetrating keratoplasty (PK) and deep anterior lamellar keratoplasty (DALK). PK involves full-thickness corneal transplantation, whereas DALK involves removing the majority of the diseased corneal tissue, leaving behind the Descemet’s membrane and corneal endothelium and then suturing the donor corneal graft (which is devoid of Descemet membrane). DALK has become more popular with surgeons in the last 10 years due to its lower rejection rate than PK [6].

We present a case of severe Pseudomonas microbial keratitis that was initially misdiagnosed as a corneal abrasion. Once appropriate topical antibiotics were given, the patient was left with dense corneal scarring and was keen on corneal graft surgery to improve vision. The patient proceeded with DALK surgery and there was premature entry into the anterior chamber intra-operatively. We discuss the surgical approach used to rectify this and continue with the planned procedure of choice rather than converting to PK and emphasise the importance of close post-operative monitoring to manage complications such as localised Descemet membrane detachment.

## Case presentation

A 32-year-old female presented to the general accident & emergency department with a one-day history of a painful red left eye after removing her soft daily-use contact lens earlier that day. She had been wearing daily contact lenses for myopic correction for many years. She denied any history of water contact or sleeping with her contact lenses on. On examination, she was found to have a small (<1 mm diameter) epithelial defect with no corneal infiltrate. She was diagnosed with a “corneal abrasion” and prescribed chloramphenicol drops to use 4x daily and referred to the ophthalmology team for a review in one week.

**Figure 1:**
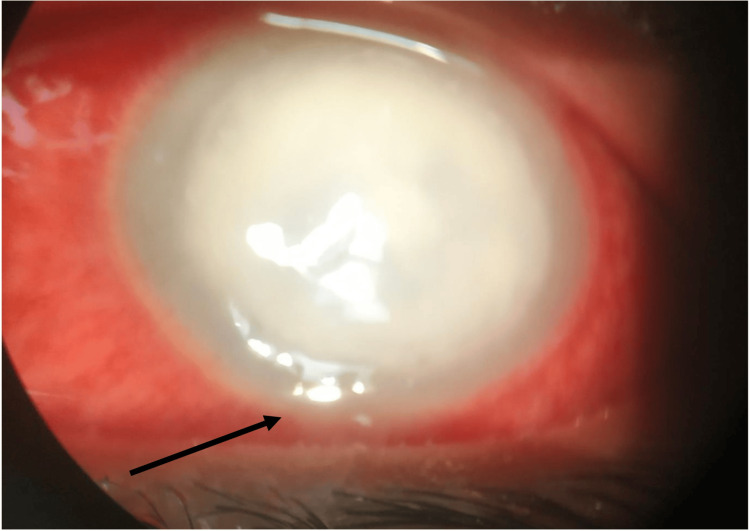
Slit-lamp photo of the left eye demonstrating the corneal abscess. The arrow highlights the formed 1 mm hypopyon in the anterior chamber.

However, she self-presented to the eye emergency department three days later with severe left eye pain, discharge, and intense photophobia. On examination, visual acuity was reduced to hand movements best corrected in the left eye. A total of 85% of the cornea was completely opaque due to a large 9 x 9 mm round abscess and a small 1 mm hypopyon was seen (Figure 1). There was evidence of stromal loss and significant peripheral corneal thinning. There was very poor view of the anterior chamber with no iris or fundal view. Ultrasound B-scan of the left eye showed no obvious posterior segment pathology. The right eye vision was 20/20 best corrected and examination was within normal limits.

Corneal specimens were obtained by scraping the base and edges of the corneal ulcer and sent for gram staining, bacterial, fungal, and Acanthamoeba cultures, and sensitivities. The clinical features were suggestive of Pseudomonas keratitis and, as a result, the patient was started on moxifloxacin 0.5% drops hourly day and night, gentamycin 1.5% drops hourly day and night, and oral doxycycline 100 mg tablets 1x/day.

The patient was reviewed daily over the next few days and reassuringly the peripheral corneal thinning stabilised. The culture results came back positive for Pseudomonas aeruginosa (sensitive to ciprofloxacin, gentamycin, and levofloxacin). Based on this, the treatment was modified to levofloxacin 0.5% drops and gentamycin 1.5% drops hourly day and night.

Over the next 14 days following treatment modification, the patient reported symptomatic improvement with resolution of pain and discharge.

**Figure 2:**
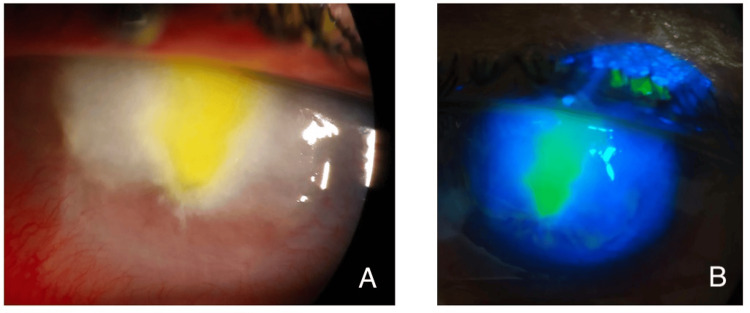
Slit-lamp photos of left eye following resolution of infection. Figure A shows central diffuse corneal scarring with inferior peripheral superficial and deep corneal vascularisation. Figure B demonstrates fluorescein staining of the same eye, with central pooling of dye.

The antibiotics were tapered over four weeks and topical dexamethasone 0.1% preservative-free was added. Over three months, the active infection resolved and there was resolution of the hypopyon and no further progression of the peripheral corneal thinning. However, the patient was left with diffuse central scarring, peripheral anterior synechiae, and four quadrants of superficial and some deep stromal corneal vascularisation (Figure 2).

The patient was lost to follow-up for 12 months as she got pregnant. She returned to see the eye department 14 months later and she was keen to consider options to improve her vision. Of note, she was breastfeeding and was not keen to take any systemic immunosuppression.

She was offered both PK and DALK and made aware of the guarded prognosis due to the deep stromal scarring. After a long discussion regarding the risks and benefits of each option, the patient opted for DALK surgery under general anaesthetic.

Pre-operative anterior segment optical coherence tomography (AS-OCT) imaging was performed and the peripheral corneal thickness was estimated at 350 μM.

During the surgery, a vacuum trephine was used, and the surgical plan was for three-quarter turns (each quarter turn is estimated to trephine the cornea by 64 μM, so 3 x 64 = 192 μM), followed by lamellar dissection.

However, there was premature entry into the anterior chamber after two quadrant turns. A decision was made to continue with the planned DALK procedure rather than converting to a PK. Nylon 10/0 interrupted sutures were placed at the site of premature entry and air was injected to re-inflate the anterior chamber. Lamellar dissection was started from the opposite side of the cornea (180 degrees away from the site of premature entry) and manual dissection was performed down to Dua’s layer. The donor corneal tissue was prepared by removing the Descemet membrane and the graft was sutured with 16 evenly distributed 10/0 nylon sutures. The anterior chamber was inflated with air, the pupil was left pharmacologically dilated, and the initial “rescue sutures” were removed at the end of the surgery.

**Figure 3:**
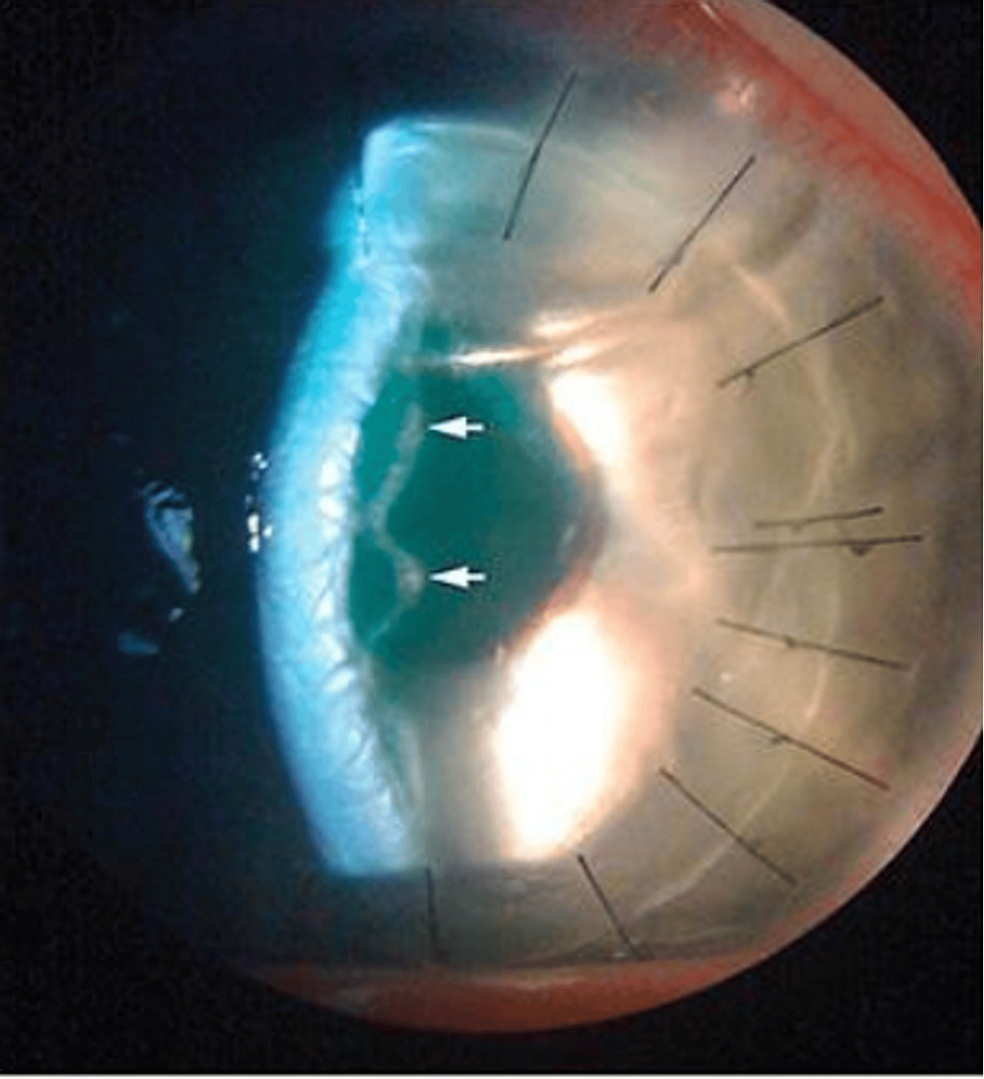
Double bubble anterior chamber sign with distinct space seen between donor stroma and host Descemet membrane.

On day one following DALK surgery, a double anterior chamber was noticed on slit-lamp examination (Figure 3), with 20% air fill.

Later that day, the patient underwent rebubbling under local anaesthetic. Two inferior sutures were removed and 100% intracameral air filling was performed before the inferior sutures were replaced. The patient was made to lay supine until the air reabsorbed.

**Figure 4:**
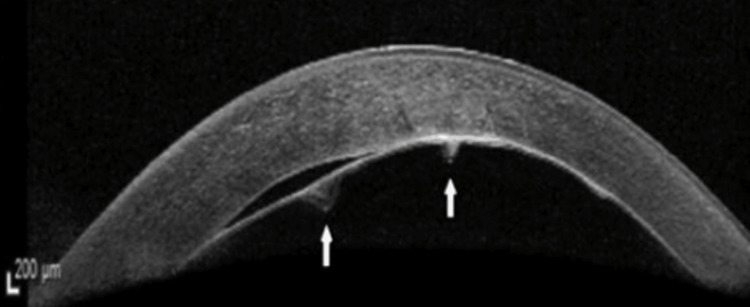
AS-OCT demonstrating a small residual “double anterior chamber”. AS-OCT: anterior segment-optical coherence tomography.

The patient was reviewed the next day and there was no obvious double bubble sign on slit-lamp examination. There was a small amount of fluid seen between the donor stroma and host Descemet membrane on AS-OCT (Figure 4) and a decision was made to monitor this.

This small area of Descemet's detachment spontaneously resolved within two weeks. The patient was referred to the contact lens department at one month and achieved the best corrected visual acuity of 20/25 (with rigid gas permeable lenses).

## Discussion

Pseudomonas keratitis is one of the most aggressive forms of bacterial keratitis and is associated with larger ulcer size [7] and significantly worse presenting visual acuity [8] than other forms of bacterial keratitis. The pathogenesis of Pseudomonas keratitis is twofold: it produces several destructive proteins [9], such as exoenzymes S, U, and elastase, and it induces an excessive inflammatory response in the cornea [9]. This excessive inflammatory response gives the clinical features of corneal thinning and perforation associated with Pseudomonas keratitis.

Different topical antibiotics are used as initial management in Pseudomonas keratitis. In some regions, monotherapy with fluoroquinolone is used. In other areas, a combination of 1.5% gentamycin and 5% cefuroxime drops is used to provide broad spectrum cover. Various randomised controlled trials have demonstrated that fluoroquinolone monotherapy is non-inferior to combination therapy [10,11]. Whilst antibiotic therapy should be initiated prior to the results of microbial cultures, it has been proven that a better therapeutic outcome is achieved when treatment can be adjusted based on culture and sensitivity results [12].

In our case, once the infection had been sterilised, the patient was left with dense stromal scarring, peripheral corneal thinning, and four-quadrant superficial and deep corneal vascularisation and was keen to consider surgical options to improve vision. Furthermore, the patient was breastfeeding and was not keen on any systemic immunosuppression. These are some of the factors that were considered in the decision-making between PK and DALK for the surgical approach. Our patient opted for DALK and the advantages of this over PK are that there is less long-term need for topical steroids [13], much lower rejection rates, and a higher chance of long-term survival [6]. However, the disadvantages of DALK are that due to the deep scarring, it is a technically more challenging surgery, and there are some intra-operative complications that can occur in DALK that may lead to the need to convert to a PK.

One of these complications (premature entry into the anterior chamber) occurred in our case despite pre-operative corneal thickness measurement with AS-OCT. This can occur in 10-26% of DALK cases [14,15]. Whilst it can be tempting to convert to a PK [16] when this occurs, this case has demonstrated that a DALK procedure can still be performed if adjustments are made. In our case, the site of premature entry was sutured and manual lamellar dissection was successfully performed from the opposite side to the site of premature entry.

Post-operatively, this patient was found to have a "double anterior chamber" sign. This is a clinical sign that describes fluid separating the host Descemet membrane from the donor stroma and occurs in 10% of cases [17]. If the area of detachment is large and central, air or gas tamponade is recommended with a high chance of reattachment with a single re-bubbling if performed within one month [18] to prevent the Descemet membrane from becoming fibrotic. Shallow detachments can often be observed and often resolve spontaneously.

## Conclusions

Pseudomonas keratitis is one of the most aggressive types of bacterial keratitis due to the intense inflammatory response that it induces in the cornea. This leads to significant corneal thinning and if not promptly and appropriately treated can lead to corneal perforation. Monotherapy with topical fluoroquinolones or combination therapy with gentamycin is recommended if Pseudomonas is suspected; however, corneal cultures and sensitivities are essential to optimise management. Various factors including the need for long-term immunosuppression, depth of corneal scarring, and patient preference are all important to consider when deciding between PK and DALK surgery for vision-impairing stromal scarring.
